# Mobility Patterns During COVID-19 Travel Restrictions in Nairobi Urban Informal Settlements: Who Is Leaving Home and Why

**DOI:** 10.1007/s11524-020-00507-w

**Published:** 2021-02-02

**Authors:** Jessie Pinchoff, Cara Kraus-Perrotta, Karen Austrian, James B. Tidwell, Timothy Abuya, Daniel Mwanga, Beth Kangwana, Rhoune Ochako, Eva Muluve, Faith Mbushi, Mercy Nzioki, Thoai D. Ngo

**Affiliations:** 1 Population Council, New York, NY USA; 2 Population Council, Nairobi, Kenya; 3 World Vision Inc, Washington, DC USA

## Abstract

Nairobi’s urban slums are ill equipped to prevent spread of the novel coronavirus disease (COVID-19) due to high population density, multigenerational families in poorly ventilated informal housing, and poor sanitation. Physical distancing policies, curfews, and a citywide lockdown were implemented in March and April 2020 resulting in sharp decreases in movement across the city. However, most people cannot afford to stay home completely (e.g., leaving daily to fetch water). If still employed, they may need to travel longer distances for work, potentially exposing them COVID-19 or contributing to its spread. We conducted a household survey across five urban slums to describe factors associated with mobility in the previous 24 h. A total of 1695 adults were interviewed, 63% female. Of these, most reported neighborhood mobility within their informal settlement (54%), 19% stayed home completely, and 27% reported long-distance mobility outside their informal settlement, mainly for work. In adjusted multinomial regression models, women were 58% more likely than men to stay home (relative risk ratio (RRR): 1.58, 95% confidence interval (CI): 1.16, 2.14) and women were 60% less likely than men to report citywide mobility (RRR: 0.40; 95% CI 0.31, 0.52). Individuals in the wealthiest quintile, particularly younger women, were most likely to not leave home at all. Those who reported citywide travel were less likely to have lost employment (RRR: 0.49; 95% CI 0.38, 0.65) and were less likely to avoid public transportation (RRR: 0.30; 95% CI 0.23, 0.39). Employment and job hunting were the main reasons for traveling outside of the slum; less than 20% report other reasons. Our findings suggest that slum residents who retain their employment are traveling larger distances across Nairobi, using public transportation, and are more likely to be male; this travel may put them at higher risk of COVID-19 infection but is necessary to maintain income. Steps to protect workers from COVID-19 both in the workplace and while in transit (including masks, hand sanitizer stations, and reduced capacity on public transportation) are critical as economic insecurity in the city increases due to COVID-19 mitigation measures. Workers must be able to commute and maintain employment to not be driven further into poverty. Additionally, to protect the majority of individuals who are only travelling locally within their settlement, mitigation measures such as making masks and handwashing stations accessible within informal settlements must also be implemented, with special attention to the burden placed on women.

## Background

Ramifications of the 2019 novel coronavirus disease (COVID-19) pandemic have been seen across the globe, and Kenya is no exception. While transmission of COVID-19 in the country remains low [1], the potential for uncontrolled outbreaks in urban slums is still of great concern given that 60–70% of Nairobi residents reside in informal settlements [2]. To prevent COVID-19, the World Health Organization has promoted physical distancing (e.g., keeping 1–2 m apart, avoiding touching) and handwashing [1], but these strategies are harder to implement in informal settlements, areas that are densely populated, overcrowded, lack access to running water or improved sanitation, are comprised of multigenerational households, have transient household members, and experience pervasive poverty with many households having unstable or informal income [3–8].

In Kenya, the first case of COVID-19 was detected on March 13, 2020, resulting in the immediate closure of schools followed by a ban on international flights and large social gatherings, including specific cultural and faith practices such as mass prayer gatherings, large weddings, and funerals, in an effort to prevent case importation from highly affected countries and super-spreading events that may accelerate transmission [9, 10]. Additionally, a nationwide dusk to dawn curfew was imposed on March 27, 2020, and a formal lockdown placed over Nairobi Metropolitan Area from April 6, 2020, which lasted for over a month. While the nationwide curfew is still scheduled to be in effect through September 2020 or longer, the lockdown over Nairobi Metropolitan Area was lifted on July 6, 2020. Policies were disproportionately focused on Nairobi as Nairobi County contains most of all of Kenya’s infections (45%) [11]. During the lockdown, the guidance was to stay home unless necessary. This guidance may be particularly challenging for informal settlement residents to follow; anecdotal evidence suggests that adherence to the mobility restrictions and curfews has been high, but enforcement may be challenging long term [12, 13]. Policies that aim to prevent the devastating health effects of COVID-19 have had the unintended consequences of severely increasing unemployment and food insecurity; those that are able to work may be forced to break social isolation to ensure their income and feed their families. While Nairobi is considered the epicenter of the pandemic in Kenya, little is known regarding the transmission dynamics within the city. Subcounty level data are available, but not for informal settlements specifically.

In general, mobility within the city of Nairobi is limited for slum residents. Car ownership among slum residents is minimal; most residents walk or rely on public transportation [14]. Motorized public transportation within slums is mainly *matatus*, privately owned and operated small 14- or 25-seater vans. Despite being widely available, *matatus* are often too costly for slum residents. Moreover, even though 68% of working adults work outside of their home slum, only about half (45%) use *matatus* as their mode of transit with most walking even over long distances [14]. Under normal circumstances, slum dwellers are not very mobile outside of their home settlement; and if they do travel, they may decide to incur the expense of using *matatus* for longer distances outside of their home settlement. During COVID-19, the Government of Kenya is requiring that public transportation vehicles including *matatus* only be filled to half capacity [15], and that everyone wear masks, resulting in increased fare costs. This may make transportation difficult for many Nairobi residents that even prior to the pandemic found these fees too high.

While the Nairobi lockdown restricted movement into and out of the city, movement within the city was not limited during the day. It is unclear how much within-city mobility was reduced because of the citywide lockdown. Those who maintained employment during the lockdown may have continued to move around the city, increasing their risk of infection by traveling on public transportation. Nationwide travel bans due to COVID-19 have been shown to result in dramatic drops in mobility (as detected by mobile phone data) in US and other high-income settings [16, 17]. A study from China suggests that complete lockdowns resulted in significant disruption of COVID-19 disease spread [18], but it is unclear what effect physical distancing measures will have in highly overcrowded, impoverished areas [19]. During the recent Ebola and Zika outbreaks, epidemiologists raised concerns about disease spread within and between informal communities due to unofficial or unreported travel [7, 20]. Travel bans and health screenings at border checkpoints in West Africa were not as effective in preventing the spread of Ebola because people often engaged in clandestine travel in order to seek employment, circumventing these mitigation policies [7].

Vulnerability and mobility are also both inextricably linked with gender. In Nairobi informal settlements, generally men are more likely than women to work. A 2004 survey of Nairobi informal settlement residents found that 73% of slum residents who work are men [14]. Only 22% of adult men and 3.6% of adult women report salaried employment [21]. Moreover, men are more likely to travel by *matatu* than women (52% of men travel by *matatu* compared to 38% of women), with this gender gap in transit choice attributed in large part to the cost [14]. Relatedly, expectations around childcare are linked with employment and transit; women with children are more likely to be unemployed, whereas the presence of children in the household did not affect men’s employment status [14]. Gender is also tied to poverty; male headed households in Nairobi informal settlements are twice as likely to transition out of poverty than female headed households [22]. Mobility and transit choice are very closely linked to income levels and employment; it is unsurprising that these link with gender [14]. During pandemics where households often face extreme economic shocks, the evidence overwhelming demonstrates that women will be disproportionately impacted. Women are more likely to be employed in informal and domestic work that cannot be transitioned remotely, and this income loss leaves women and girls incredibly vulnerable to food insecurity, violence, and other adverse outcomes [23].

In March 2020, the Kenyan Ministry of Health COVID-19 Taskforce partnered with the Population Council to conduct a series of mobile phone surveys to assess knowledge, attitudes, practices, and needs related to COVID-19 among residents of five informal settlements across Nairobi. The third round of the survey, conducted in May, also examined mobility patterns of respondents. In the previous 24 h, most respondents left their home at least once but only traveled locally; a small proportion of respondents stayed at home, while the rest traveled longer distances. We explore the demographics, employment status, and risk perceptions of different groups to understand who was traveling across the city and why. Understanding mobility dynamics in informal settlements will help policymakers design strategic interventions and approaches to protect their communities from COVID-19 and future public health crises.

## Methods

The study methods have been described elsewhere [24]. In brief, the Population Council has collaborated with the Kenyan Ministry of Health COVID-19 Taskforce to conduct a series of mobile phone–based surveys with a sample of households across five urban slums in Nairobi. Participants were randomly selected from two ongoing Population Council cohort studies: The Adolescent Girls Initiative-Kenya (AGI-K) study in Kibera and Huruma slums, and the NISITU study based in Kariobangi, Dandora, and Mathare slums. Phone numbers for households from AGI-K and NISITU were up to date. The COVID-19 cohort is comprised of a stratified sample of 2009 households selected from these five slums.

Due to the restriction of movement in Nairobi and physical distancing policies to reduce spread of COVID-19, all data collection was conducted by mobile phone. A short questionnaire was developed and conducted in March 2020. Subsequent surveys with modified or adapted questions were conducted in April, May, and June 2020. This analysis uses the May 2020 survey results (round 3), linked with demographic information collected in round 1. The May survey included questions on demographics, knowledge, and attitudes regarding COVID-19, preventive behaviors such as wearing a mask, and social and economic effects such as losing full or partial income or skipping meals.

Data collection was carried out by 77 Kenyan enumerators who were trained on conducting mobile phone surveys, and before each new survey round, a review training session was held to review the new questions. The March survey included questions on basic demographics, awareness of COVID-19, knowledge, perceived risk, preventive behaviors being implemented, channels of information, and trustworthiness of each source. In round 2 (April), questions regarding social and economic effects on households were added, for example, loss of employment, skipping meals, household costs, and gender-based violence or tensions experienced in the household. In round 3 (May), more detailed questions were added, for example how often participants are skipping meals. Each surveyor completed 10–20 surveys per day, and all phone numbers were tried up to three times if not reached on the first attempt. This analysis focuses on round 3 survey responses regarding mobility patterns. The survey was collected using Open Data Kit and exported to Stata v15 for analysis.

## Ethical Approval

We received ethical approval for the initial AGI-K and NISITU protocols from Population Council IRB (p661 and p829) and local Kenyan ethical approval through AMREF ESRC (P143/2014 and P407/2017). The Ministry of Health provided written approval to conduct the surveys with these cohorts. All personally identifiable information was removed to ensure confidentiality; each household received a coded ID number. Participants were told they could terminate the survey at any time or refuse to answer specific questions. Participants were informed beforehand that they would be paid 100 Kenyan shillings (~ US$1) for their time (transferred via mPesa mobile money).

### Data Analysis

A variable for mobility was created from multiple survey responses. Participants were asked if they had left home in the previous 24 h at all; if so, how many times; and then separately, of these trips out of the home how many were outside of their informal settlement. Lastly, participants were asked the reason for their travel outside of their informal settlement. Triangulating these responses, we created a three-category mobility variable: (1) did not leave home, (2) neighborhood mobility (left home but stayed nearby), and (3) city-wide mobility (when left home, left informal settlement).

Basic respondent characteristics, such as demographics, perceived risk of COVID-19, and social and economic effects of COVID-19 including loss of employment, were tabulated by the three-level mobility variable. We tested but did not include a marital status variable because by age 24, almost 100% of men were married. Multinomial logistic regression models were then fit to compare the factors associated with each mobility category, including adjustment for informal settlement of residence. These models were then run stratified by gender to explore variation within male and female respondents.

## Results

A total of 1695 adults across five urban informal settlements were interviewed in round 3 of the COVID-19 survey. Overall, 63% were female, 59% were married, and 42% had experienced a complete loss of income due to COVID-19. There was variation in demographic characteristics by mobility category (Table 1). Most participants (54%) reported neighborhood mobility. Of these, 68% were female, 46% had fully lost employment due to COVID-19. About 19% of participants had not left home at all in the previous 24 h; these were mostly female (77%) and had completely lost employment (57%). About 27% of respondents reported citywide mobility, these were mostly men (66% male) and about quarter (26%) had lost full employment. Relatedly, most of those who stayed home or reported neighborhood mobility (81% and 82%, respectively) said they had avoided public transportation due to COVID-19, whereas only 56% of those reporting citywide mobility were able to avoid public transportation. Of participants who reported citywide mobility, over half said the main reason was for work, and over 20% said for job hunting. Less than 15% cited shopping for things unavailable closer to home, to see friends or seek entertainment, or for health services. There was some variation by informal settlement, described in an Appendix; for example, respondents in Huruma were most likely to report staying home completely (25%; *p* < 0.001), and residents of Dandora and Mathare settlements reporting more citywide mobility (32% each; *p* < 0.001) (Appendix 1).

**Table 1:** Characteristics of respondents by reported mobility in the last 24 h

	Total	Neighborhood mobility	No mobility (stayed home)	Citywide mobility	*p* value
	*N* = 1695	*N* = 915	*N* = 328	*N* = 452	
Female	1075 (63%)	625 (68%)	252 (77%)	198 (44%)	< 0.001
Age (in categories)					0.34
< 24 years	348 (21%)	193 (21%)	78 (24%)	77 (17%)	
25–34 years	321 (19%)	172 (19%)	57 (17%)	92 (20%)	
35–44 years	619 (37%)	325 (36%)	121 (37%)	173 (38%)	
45 + years	407 (24%)	225 (25%)	72 (22%)	110 (24%)	
Married					0.038
Married or living together	996 (59%)	530 (58%)	176 (54%)	290 (64%)	
Single	407 (24%)	218 (24%)	92 (28%)	97 (21%)	
Divorced/separated/widowed	288 (17%)	165 (18%)	59 (18%)	64 (14%)	
Household location					< 0.001
Kibera	381 (22%)	231 (25%)	73 (22%)	77 (17%)	
Dandora	410 (24%)	214 (23%)	82 (25%)	114 (25%)	
Huruma	220 (13%)	121 (13%)	54 (16%)	45 (10%)	
Kariobangi	330 (19%)	158 (17%)	67 (20%)	105 (23%)	
Mathare	352 (21%)	190 (21%)	51 (16%)	111 (25%)	
Wealth category quintile					0.004
Poorest	346 (20%)	207 (23%)	69 (21%)	70 (15%)	
Poorer	334 (20%)	200 (22%)	63 (19%)	71 (16%)	
Moderate	350 (21%)	176 (19%)	65 (20%)	109 (24%)	
Wealthier	548 (32%)	274 (30%)	109 (33%)	165 (37%)	
Wealthiest	117 (7%)	58 (6%)	22 (7%)	37 (8%)	
Perceived risk of COVID-19					0.008
No/low risk	466 (27%)	240 (26%)	113 (34%)	113 (25%)	
Medium risk	491 (29%)	255 (28%)	91 (28%)	145 (32%)	
High risk	722 (43%)	412 (45%)	119 (36%)	191 (42%)	
Complete loss of income	720 (42%)	417 (46%)	187 (57%)	116 (26%)	< 0.001
Partial loss of income	711 (42%)	367 (40%)	82 (25%)	262 (58%)	< 0.001
Avoid public transportation	1272 (75%)	753 (82%)	267 (81%)	252 (56%)	< 0.001
Mean (std) number of trips	2.77 (2.4)	0 (0)	2.8 (2.4)	2.7 (2.4)	*p* = 0.145

In multinomial regression models, we explored the association between mobility, loss of employment, and gender. Compared with those who reported neighborhood mobility, those who did not leave home at all were almost twice as likely to be female (Relative Risk Ratio (RRR) = 1.58; 95% confidence interval (CI): 1.16, 2.14), and 1.71 times more likely to have lost full employment due to COVID-19 (RRR = 1.71; 95% CI: 1.31, 2.24) (Table 2). They perceived they were not at high risk of contracting COVID-19. Those who did not leave home compared with those who reported neighborhood mobility were also more likely to be wealthy. Comparing those who reported citywide mobility to those reported neighborhood mobility, they were 0.40 times less likely to be female (RRR = 0.40; 95% CI: 0.31, 0.52) and 0.49 times less likely to have lost full employment (RRR = 0.49; 95% CI: 0.38, 0.65). Participants who reported citywide mobility perceived themselves to be at medium risk of contracting COVID-19 and were significantly less likely to say they avoided public transportation in the last 2 weeks.

**Table 2:** Multinomial logistic regression analysis of factors associated with mobility category

	Neighborhood mobility	No mobility (stayed home)	Citywide mobility
Variables	RRR 95% CI	RRR 95% CI	RRR 95% CI
Female (vs male) gender		1.577**	0.402**
	REF	(1.159—2.144)	(0.312—0.519)
Age category			
35–44 years (REF)	REF	REF	REF
< 24 years		1.213	0.579**
		(0.852—1.728)	(0.406—0.826)
25–34 years		0.870	0.987
		(0.597—1.269)	(0.700—1.390)
45 + years		0.937	0.810
		(0.661—1.329)	(0.585—1.119)
Quintiles of wealth			
Poorest (REF)	REF	REF	REF
Poorer		1.088	0.826
		(0.685—1.728)	(0.508—1.343)
Middle		2.896**	1.184
		(1.294—6.480)	(0.572—2.451)
Wealthier		3.065**	1.251
		(1.350—6.959)	(0.601—2.605)
Wealthiest		2.577*	1.097
		(1.002—6.623)	(0.476—2.531)
Perceived risk of COVID-19			
Low/no risk (REF)	REF	REF	REF
Medium risk		0.754	1.330
		(0.540—1.053)	(0.959—1.844)
High risk		0.615**	0.848
		(0.451—0.838)	(0.624—1.152)
Complete loss of income	REF	1.713**	0.494**
		(1.311—2.237)	(0.378—0.645)
Avoid public transportation due to COVID-19	REF	0.816	0.296**
		(0.581—1.147)	(0.226—0.388)
Informal settlement			
Kibera (REF)	REF	REF	REF
Dandora		0.461	1.175
		(0.203—1.045)	(0.565—2.441)
Huruma		1.207	1.445
		(0.754—1.930)	(0.877—2.380)
Kariobangi		0.561	1.608
		(0.254—1.238)	(0.793—3.261)
Mathare		0.346**	1.562
		(0.156—0.768)	(0.785—3.109)

^**^p < 0.01, * *p* < 0.05

In multinomial regression models stratified by gender, differing associations emerged. Among men, 35–44 years olds were more likely to stay home completely, compared to men ages < 24, 25–34 or 45 + year olds (Table 3). They also perceived themselves to be at less risk of COVID-19 compared to men who reported neighborhood mobility. Among men who reported citywide mobility, compared to neighborhood mobility, these men were 0.40 times as likely as having lost full employment (RRR = 0.40, 95% CI: 0.27, 0.61) and were 0.24 times less likely to report avoiding public transportation (RRR = 0.24; 95% CI: 0.16, 0.37) (Table 3). Among women, the main characteristics associated with staying home completely compared to neighborhood mobility were age < 24 years, being in the wealthiest quintile, and having lost full employment. Women who stayed home completely perceived their risk of contracting COVID-19 to be lower than women who report neighborhood mobility. Women who report citywide mobility were less likely to have lost employment and less able to avoid public transportation.

**Table 3:** Multinomial logistic regression model of factors associated with mobility category stratified by gender

	Male respondents			Female respondents		
	Neighborhood mobility	No mobility (stayed home)	Citywide mobility	Neighborhood mobility	No mobility (stayed home)	Citywide mobility
Variables	RRR 95% CI	RRR 95% CI	RRR 95% CI	RRR 95% CI	RRR 95% CI	RRR 95% CI
Age category						
35–44 years (REF)	REF	REF	REF	REF	REF	REF
< 24 years		0.533	0.547*		1.701*	0.565*
		(0.266—1.066)	(0.322—0.931)		(1.113—2.600)	(0.337—0.949)
25–34 years		0.420	0.871		1.087	1.056
		(0.168—1.049)	(0.488—1.556)		(0.712—1.659)	(0.686—1.624)
45 + years		0.442*	0.758		1.202	0.766
		(0.219—0.893)	(0.467—1.230)		(0.799—1.808)	(0.482—1.215)
Quintiles of wealth						
Poorest (REF)	REF	REF	REF	REF	REF	REF
Poorer		0.854	0.627		1.200	0.935
		(0.280—2.606)	(0.270—1.456)		(0.718—2.005)	(0.510—1.715)
Middle		1.819	0.851		3.485**	1.502
		(0.321—10.314)	(0.266—2.724)		(1.387—8.758)	(0.571—3.953)
Wealthier		2.198	0.881		3.746**	1.726
		(0.383—12.612)	(0.272—2.854)		(1.455—9.643)	(0.650—4.583)
Wealthiest		1.111	0.757		3.804*	1.486
		(0.158—7.809)	(0.210—2.722)		(1.264—11.451)	(0.461—4.789)
Perceived risk of COVID-19						
Low/no risk (REF)	REF	REF	REF	REF	REF	REF
Medium risk		0.891	1.450		0.723	1.325
		(0.453—1.750)	(0.873—2.409)		(0.490—1.066)	(0.849—2.069)
High risk		0.489*	0.696		0.675*	1.000
		(0.263—0.909)	(0.442—1.098)		(0.470—0.971)	(0.652—1.534)
Complete loss of income		1.293	0.404**		1.902**	0.548**
		(0.765—2.187)	(0.270—0.605)		(1.386—2.609)	(0.381—0.787)
Avoid public transportation due to COVID-19		0.687	0.244**		0.853	0.336**
		(0.368—1.284)	(0.162—0.366)		(0.567—1.284)	(0.231—0.488)
Informal settlement						
Kibera (REF)	REF	REF	REF	REF	REF	REF
Dandora		0.734	1.673		0.366*	0.784
		(0.132—4.092)	(0.529—5.288)		(0.141—0.945)	(0.289—2.126)
Huruma		1.297	1.311		1.119	1.482
		(0.416—4.045)	(0.542—3.168)		(0.665—1.884)	(0.801—2.740)
Kariobangi		0.802	1.656		0.498	1.651
		(0.148—4.351)	(0.537—5.106)		(0.201—1.234)	(0.646—4.218)
Mathare		0.543	2.174		0.265**	1.182
		(0.100—2.948)	(0.723—6.534)		(0.106—0.664)	(0.471—2.966)

^**^p < 0.01, *p < 0.05

## Discussion

Overall, we found variation in mobility patterns for individuals residing in urban slums across Nairobi, 3 months into the COVID-19 pandemic. Government promotion of physical distancing and staying at home to reduce COVID-19 risk resulted in many business closures and significant economic insecurity. We found that only a very small number of survey respondents could stay at home entirely, mainly young, unemployed female respondents from higher wealth quintile households. These respondents are at the lowest risk of COVID-19 transmission since they can reduce their interactions to only household contacts. Most respondents, however, report mobility within their informal settlement, likely for sanitation needs, fetching water, and other necessities, with most reporting one to two local trips per day. A third category is those who travel around Nairobi, mainly males traveling for work, likely using public transportation. They are at increased risk of COVID-19 transmission as they interact with others in transit and in workplaces. Tailored policies may be useful to address the needs of those traveling locally for essential needs, compared to those traveling longer distances to maintain employment.

Critical attention must be given to the subgroup that reports citywide mobility, mainly for work or job seeking. Our cohort already shows alarmingly high rates of unemployment only 3 months into the crisis, coupled with increased rates of food insecurity, rising costs for household necessities, and challenges paying fees for health services [24]. Households in urban informal settlements or slums are vulnerable to economic shocks including effects of COVID-19 mitigation measures as they rely primarily on unstable forms of income [21]. Our study finds that men who have retained their employment are more likely to report citywide mobility. From riding public transportation and interacting with others at work sites or offices, these respondents are potentially increasing their risk of infection and contributing to spread of COVID-19 around Nairobi. These respondents did perceive their risk to be high, but food insecurity and hunger in these precarious conditions may exceed the threat of COVID-19 infection [25]. Our findings, along with recent evidence from Quaife et al. [12] that men have more social contacts than women, may partially explain the differences in behavior that puts men at higher risk for contracting COVID-19. More research is required on how to reduce risk of transmission on public transportation where masks and distancing are suggested but likely challenging to enforce.

The least at-risk group we identified is a small subset of individuals that were able to fully stay home in the last 24 h. These were mainly young women who had lost employment, and who resided in households with the highest wealth quintiles. This is consistent with findings from the USA that found higher income groups reduced mobility quicker than low income groups [26]. This is likely tied to employment; essential workers or healthcare providers were forced to travel and continue working outside the home. A recent study in Nairobi also found that as socioeconomic status increases, the number of contacts decreases, likely relating to less COVID-19 transmission [12]. The gender inequity is in line with research on employment in slums; Emina et al. [21] found that women are less likely to be employed at all (51% economically inactive), and if they are employed, it is most likely casual employment (21%) or unestablished business (13%). These younger women are at low risk because they stay home, but note, this does not mean that everyone in their household is able to stay home. Likely a parent or spouse is still going out to collect necessary items, so some risk of COVID-19 infection still exists. Overall, higher wealth may be providing some protection to households that are able to minimize contact and trips outside the home and reduce their risk of COVID-19 exposure.

However, the most reported pattern was neighborhood mobility, with over half of our respondents reporting they left home 1–2 times in the previous 24 h but staying within their informal settlement. Challenges in informal settlements to adhere to strict physical distancing and stay at home guidance include the need to fetch water, collect food and fuel, and access sanitation and hygiene facilities including latrines that are not located inside each home. With low availability of piped water, many toilets serving multiple household units, and lack of refrigeration or electricity to store groceries, most people will have to leave home at least once per day [25]. Increasing safety measures at key points such as water pumps and local market stalls is critical, for example, reducing crowding at these locations, and making hand sanitizer or soap available. Promoting wearing masks, making sanitizer or water available, and taking steps to reduce crowding at key choke points (e.g., a water pump) will help reduce the number of social interactions and contacts and make them safer when they do occur. There was some variation in mobility by informal settlement, suggesting that additional geographic targeting of interventions may be useful. For example, the highest proportion of respondents reporting no mobility were from Huruma settlement, which may be related to better infrastructure and higher quality housing (multistory, more permanent buildings). Understanding the infrastructural variation between informal settlements and other communities across cities may be useful to understand within-city variation in exposure to COVID-19 as well as ability to adopt promoted preventive behaviors (for example, washing hands with water and soap is only possible if water is accessible).

This study has several limitations. First, we conducted our sampling based on a listing of households with an adolescent residing there; therefore, it is not fully representative of all households in informal settlements. For example, households with only an elderly couple or where a divorced man lives alone would not be eligible for inclusion. We also did not ask about mobility prior to COVID-19, so our questions are subjective regarding changes in mobility as perceived by the respondent. Second, we are missing some information on reasons for neighborhood mobility. While we asked where people were going for citywide mobility, we did not ask for details if the movement was within their neighborhood. We assume this is for accessing sanitation facilities, fetching water, and purchasing basic necessities, but this could include nonessential trips such as socializing or seeking entertainment. Lastly, a major limitation is that most surveys were conducted over the weekend. We do see significant difference in mobility by day of the week, but due to the small number of interviews on weekdays, we could not adjust for this in our models. Available surveys suggest that neighborhood mobility is higher on weekends than weekdays, while citywide mobility is higher on weekdays—likely because it is related to employment. In future studies, more weekday surveys would be important to understand patterns of commuting for work compared to other reasons.

Nairobi implemented curfews and a city containment policy, meaning mobility within Nairobi was allowed, but not from the city to other parts of the country. This type of less restrictive lockdown was necessary for a place like Nairobi where people must leave home daily for basic needs and may need to travel around the city for work. Even without strict enforcement, most travel outside of one’s informal settlement was only for work or job hunting; less than 15% of respondents that reported citywide mobility said it was for social reasons or seeking healthcare. To better protect these residents, research on how to increase adoption of preventive behaviors on public transportation (e.g., wearing a mask, opening windows, or leaving seats empty) is needed. Additionally, more in-depth research on why residents are leaving home, with attention to gender specific needs, could help policymakers identify targeted opportunities for intervention to reduce transmission of COVID-19 within informal settlements, for example, promotion of physical distancing at water pumps or distribution of masks at local markets or on *matatus*. Because economic insecurities preclude slums residents from staying home completely, it is critical to develop tailored policies and programs so that they can travel safely and reduce the spread of COVID-19. Additional resources and support to increase the option of sheltering in place, particularly for high-risk individuals could also be promoted, discouraging unnecessary citywide travel and interactions.

## Appendix A

**Table 4:** Participant characteristics by informal settlement of residence (Kibera, Dandora, Huruma, Kariobangi, or Mathare) in Nairobi, Kenya

	Kibera	Dandora	Huruma	Kariobangi	Mathare	Total	*p* value
	*N* = 381	*N* = 410	*N* = 220	*N* = 330	*N* = 352	*N* = 1693	
Gender							< 0.001
Male	100 (26%)	190 (46%)	45 (20%)	154 (47%)	131 (37%)	620 (37%)	
Female	281 (74%)	220 (54%)	175 (80%)	176 (53%)	221 (63%)	1073 (63%)	
Age (in categories)							< 0.001
< 24 years	52 (14%)	107 (26%)	39 (18%)	100 (30%)	49 (14%)	347 (20%)	
25–34 years	57 (15%)	91 (22%)	28 (13%)	57 (17%)	88 (25%)	321 (19%)	
35_44 years	152 (40%)	142 (35%)	89 (40%)	97 (29%)	139 (39%)	619 (37%)	
45 + years	120 (31%)	70 (17%)	64 (29%)	76 (23%)	76 (22%)	406 (24%)	
Wealth quantile							< 0.001
Poorest	277 (73%)	0 (0%)	67 (30%)	0 (0%)	0 (0%)	344 (20%)	
Poorer	101 (27%)	9 (2%)	145 (66%)	34 (10%)	45 (13%)	334 (20%)	
Middle	3 (1%)	75 (18%)	8 (4%)	103 (31%)	161 (46%)	350 (21%)	
Wealthier	0 (0%)	273 (67%)	0 (0%)	152 (46%)	123 (35%)	548 (32%)	
Wealthiest	0 (0%)	53 (13%)	0 (0%)	41 (12%)	23 (7%)	117 (7%)	
Perceived risk of COVID-19							0.033
No/low risk	119 (31%)	109 (27%)	72 (33%)	88 (27%)	76 (22%)	464 (27%)	
Medium risk	112 (29%)	114 (28%)	68 (31%)	95 (29%)	102 (29%)	491 (29%)	
High risk	146 (38%)	185 (45%)	78 (35%)	144 (44%)	169 (48%)	722 (43%)	
Complete loss of income							0.066
No	219 (57%)	233 (57%)	118 (54%)	212 (64%)	192 (55%)	974 (58%)	
Yes	162 (43%)	177 (43%)	102 (46%)	118 (36%)	160 (45%)	719 (42%)	
Partial loss of income							0.095
No	219 (57%)	258 (63%)	128 (58%)	174 (53%)	203 (58%)	982 (58%)	
Yes	162 (43%)	152 (37%)	92 (42%)	156 (47%)	149 (42%)	711 (42%)	
Avoid public transportation due to COVID-19							0.24
No	106 (28%)	111 (27%)	47 (21%)	74 (22%)	84 (24%)	422 (25%)	
Yes	275 (72%)	299 (73%)	173 (79%)	256 (78%)	268 (76%)	1,271 (75%)	
Mobility category							< 0.001
Neighborhood mobility	231 (61%)	214 (52%)	121 (55%)	158 (48%)	190 (54%)	914 (54%)	
No mobility (stayed home)	73 (19%)	82 (20%)	54 (25%)	67 (20%)	51 (14%)	327 (19%)	
Citywide mobility	77 (20%)	114 (28%)	45 (20%)	105 (32%)	111 (32%)	452 (27%)	
